# The relationship between serum albumin and prostate-specific antigen: A analysis of the National Health and Nutrition Examination Survey, 2003–2010

**DOI:** 10.3389/fpubh.2023.1078280

**Published:** 2023-03-06

**Authors:** Kailiang Xu, Youji Yan, Cong Cheng, Shiqin Li, Yixiang Liao, Jinmin Zeng, Zhongjun Chen, Jiajie Zhou

**Affiliations:** Department of Urology, Jingzhou Central Hospital, Jingzhou Hospital Affiliated to Yangtze University, Jingzhou, China

**Keywords:** serum albumin, prostate-specific antigen (PSA), NHANES database, non-linear relationship, inflection point

## Abstract

**Background:**

Previous studies have shown that serum albumin is associated with prostate cancer (PCa), but not with prostate-specific antigen (PSA) levels in populations without PCa history. Therefore, we analyzed secondary data provided by the National Health and Nutrition Examination Survey (NHANES) (2003–2010).

**Methods:**

In total, 5,469 participants were selected from the NHANES database (2003–2010). Serum albumin and PSA levels were serially considered independent and dependent variables, serially. A number of covariates were included in this study, including demographic, dietary, physical examination, and comorbidity data. Using weighted linear regression model and smooth curve fitting, the linear and non-linear relationship between serum albumin and PSA was investigated.

**Results:**

After modulating underlying interference factors, the weighted multivariate linear regression analysis revealed that serum albumin did not independently predict PSA levels (β = −0.009 95%CI: −0.020, 0.002). Nevertheless, a non-linear relationship was found between serum albumin and PSA, with a point of 41 g/L. Left of the inflection point, the effect size, 95%CI, and *P*-value were 0.019 (log2 transformation) (−0.006, 0.043) and 0.1335, respectively. We found a negative association between serum albumin and PSA on the right side of the inflection point, with effect size, 95%CI, and a *P*-value of −0.022 (log2 transformation) (−0.037, −0.007), 0.0036.

**Conclusion:**

In summary, serum albumin and PSA levels are not linearly related. When serum albumin levels exceed 41 g, serum albumin levels are negatively associated with PSA levels.

## Introduction

Prostate cancer (PCa) remains to be the most diagnosed cancer in men and the second leading cause of cancer-related death worldwide (1). In the United States, the estimated number of new cancer cases and deaths caused by PCa was 268,490 and 34,500 in 2022 (2). Different countries have varying rates of PCa. For example, the incidence rate of Chinese and African American men is 0.8 and 102.1 per 100,000, severally (3). Nevertheless, the rising economic growth, longer life expectancy, and cultural exchange have significantly contributed to the increase in incidence among the Chinese (4, 5). A growing body of evidence suggests that protein-limitation diets are associated with lower PCa rates and sufficient lycopene intake could be protective against the high risk of PCa in the Non-Hispanic White men (6, 7).

As the global incidence of PCa increases, it is important to improve its early detection and diagnosis to reduce the death rates. For the first time in 1980, Papsidero et al. quantified PSA levels in human blood, signaling the clinical application of PSA, which has since become a common screening tool for PCa today (8–10). Bergengren et al. revealed that using PSA testing resulted in a 15% decrease in prostate cancer deaths from 1996 to 2016 (11) and it is crucial to clarify the factors that affect PSA in order to ensure quality screening and prevent missed diagnoses. According to recent studies, a higher level of folate may reduce PSA levels and sugar consumption is positively and independently linked to PSA levels in adult American males without prostate cancer (12, 13). The dietary protein intake positively correlated with increased PSA levels when it exceeded the threshold of 181.8 g and over 1,151 mg of dietary phosphorus per day may increase PSA levels (14, 15). However, the relationship between PSA levels and serum albumin remains unknown. Therefore, we performed a secondary analysis aimed at investigating the relationship between serum albumin and PSA levels using NHANES data. Furthermore, we assessed whether PSA levels would decrease or increase in response to serum albumin changes.

## Methods

### Data source

The NHANES, headed by the National Centers for Disease Control (CDC) and Prevention National Health Statistics Center, was a research program projected to assess the health and nutrition of adults and children in America. Research ethics review board approval was granted to the NHANES protocol by National Center for Health Statistics research ethics review board. Informed consent was acquired from all participants. The comprehensive introduction can be download from the CDC official website (www.cdc.gov/nchs/nhanes/tutorials/default.asp). In our study, we collected data from the 2003–2010 NHANES datasets (Including five circles).

### Study population

**Figure 1:**
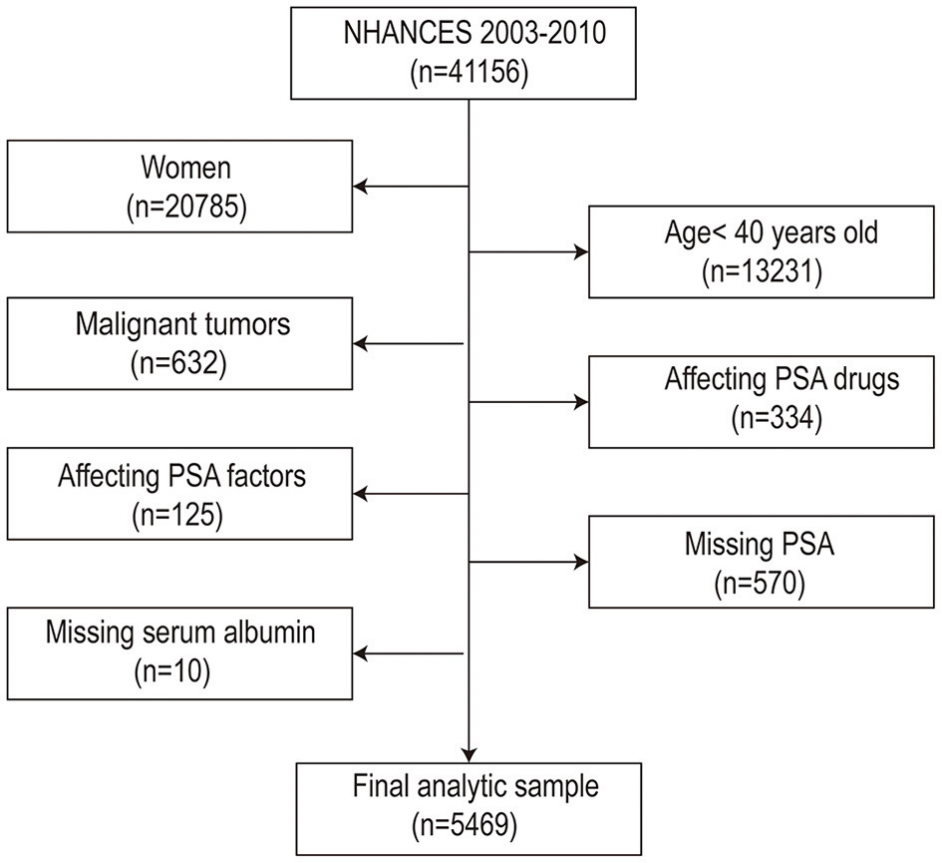
Flow chart of procedures from identification of eligible patients to final inclusions.

Throughout this study, 41,156 participants initially participated in NHANES between 2003 and 2010. Several elimination screenings were conducted, out of which 5,469 men were eventually enrolled for data analysis. The filter criteria were as follows: (1) Female (*n* =20,371); (2) Males < 40 years old (*n* = 7,140); (3) Male tumor patients (*n* = 6,508); (4) Influencing PSA drugs: Men who used 5ARI or other forms of hormone therapy and drugs (*n* = 6,174); (5) Affecting PSA factors: Men who had prostatitis or recent prostate operations (i.e., rectal examination within 1 week); surgery, cystoscopy within 1 month; Prostate biopsy (*n* = 6,049); (6) PSA data was missing (*n* = 5,479); (7) Albumin data was missing (*n* = 5,469). Finally, 5,469 research objects were included in the research (the flow chart was comprehensively presented in Figure 1). Informed consent was obtained from all participants before the interview and inspection took place.

### Variables

The dependent variable was serum albumin (g/L), whereas independent variable was serum PSA (ng/ml). A description of serum albumin and PSA measurement is available on NHANES official website (https://www.cdc.gov/nchs/nhanes). We selected covariates that had been confirmed to be associated with serum albumin and/or PSA levels in previous studies. The covariates were as follows: categorical variables included race, education level, diabetes-history, failing kidney-history, emphysema-history, stroke-history, marital status-history, coronary heart disease-history, hypertension-history and asthma-history. The continuous variables included Age (year), Poverty income ratio, Serum glucose (mmol/L), Body mass index (kg/m^2^), Creatinine (umol/L), Aspartate aminotransferase (U/L), Alanine aminotransferase (U/L), Cholesterol (mmol/L), Blood urea nitrogen (mmol/L), Lactic dehydrogenase (U/L), Serum uric acid (umol/L), and Triglycerides (mmol/L). Generally, in the NHANES database, covariates originated from demographic, dietary, examination, laboratory, and questionnaire data, allowing us to comprehensively examine the variables.

### Statistical analysis

All estimates took into account NHANES sample weights. Because PSA was skewed, we transformed it using the log2 function. Our statistical analyses included three steps. First, four groups were established based on serum albumin levels (quartiles). Frequencies or percentages were used to express categorical variables, whereas means ± standard deviations were applied to express continuous variables. Secondly, the weighted univariate and multivariate linear regression model was employed: model I was run without adjusting for covariates; model II was run with only socio-demographic data adjusted; and model III was run with model 2 plus other covariates as shown in Table 1. As a third step in the data analysis, smooth curve fitting was used to examine the non-linearity between serum albumin and PSA levels. Additionally, the threshold effect of serum albumin and PSA levels was examined using two-piecewise linear regression models. Statistical software R (http://www.r-project.org, The R Foundation) and EmpowerStats (http://www.empower-stats.com, X&Y Solutions, Inc., Boston, MA) were used to perform the analysis. Statistical significance was defined as a *p*-value < 0.05 (two-sided).

**Table 1:** Baseline characteristics of selected participants.

**Serum albumin (g/L)**	**Q1 (19.00–40.00)**	**Q2 (41.00–42.00)**	**Q3 (43.00–44.00)**	**Q4 (45.00–53.00)**	***P-*** **value**
Total prostate specific antigen (ng/mL). log2 transform	0.01 ± 1.39	−0.03 ± 1.25	−0.06 ± 1.16	−0.19 ± 1.11	<0.0001
Age	58.63 ± 12.01	55.77 ± 11.28	54.13 ± 10.76	52.55 ± 9.60	<0.0001
Ratio of family income to poverty	2.91 ± 1.56	3.19 ± 1.53	3.41 ± 1.53	3.46 ± 1.50	<0.0001
Race/ethnicity (%)					<0.0001
Mexican American	6.66	7.18	7.28	5.58	
Other Hispanic	3.53	3.43	3.97	2.95	
Non-Hispanic White	68.80	72.76	76.06	79.01	
Non-Hispanic Black	16.27	11.25	8.15	6.11	
Other race—including multi-racial	4.74	5.38	4.54	6.34	
Education level (%)					<0.0001
Less than high school	25.29	20.74	17.07	15.16	
High school	28.03	26.31	23.77	24.56	
More than high school	46.19	52.92	59.16	60.28	
Marital status (%)					0.0002
Married	65.93	70.07	72.35	73.77	
Single	27.96	23.60	22.62	21.41	
Living with partner	5.78	6.34	4.92	4.38	
Serum glucose (mmol/L).	6.28 ± 2.70	5.94 ± 2.24	5.65 ± 1.93	5.48 ± 1.43	<0.0001
Body mass index (kg/m^2^)	30.74 ± 7.51	29.64 ± 5.61	28.84 ± 4.96	27.84 ± 4.50	<0.0001
Creatinine (ummol/L)	97.71 ± 61.69	90.01 ± 36.50	89.23 ± 20.42	88.71 ± 22.01	<0.0001
Aspartate aminotransferase (U/L)	28.71 ± 21.27	27.72 ± 14.12	27.33 ± 11.89	28.58 ± 11.65	0.0338
Alanine aminotransferase (U/L)	28.84 ± 29.33	29.67 ± 18.86	29.63 ± 15.23	31.41 ± 17.00	0.0049
Cholesterol (mmol/L)	4.88 ± 1.15	5.17 ± 1.13	5.27 ± 1.03	5.42 ± 1.07	<0.0001
Blood urea nitrogen (mmol/L)	5.36 ± 2.65	5.01 ± 1.87	5.08 ± 1.73	5.04 ± 1.74	<0.0001
Lactic dehydrogenase (U/L)	135.47 ± 32.11	130.76 ± 26.61	129.57 ± 24.38	131.60 ± 25.63	<0.0001
Serum uric acid (umol/L)	365.12 ± 85.96	359.22 ± 74.96	365.96 ± 74.20	362.70 ± 71.69	0.1068
Triglycerides (mmol/L)	1.95 ± 1.91	2.06 ± 1.77	2.01 ± 1.55	2.09 ± 1.65	0.2043
Hypertension history (%)					<0.0001
Yes	46.39	41.00	37.61	33.90	
No	53.36	58.90	62.39	65.72	
Failing kidney history (%)					<0.0001
Yes	4.86	1.99	1.07	1.11	
No	94.77	97.85	98.88	98.83	
Diabetes history (%)					<0.0001
Yes	18.33	13.22	9.52	7.64	
No	78.96	84.65	88.48	89.89	
Borderline	2.64	2.10	1.95	2.45	
Stroke history (%)					<0.0001
Yes	5.61	3.74	2.14	2.14	
No	94.00	96.07	97.56	97.64	
Asthma history (%)					0.0051
Yes	13.10	9.42	9.39	9.18	
No	86.61	90.32	90.28	90.82	
Emphysema history (%)					<0.0001
Yes	4.73	1.82	2.00	1.59	
No	94.78	97.69	97.94	98.41	
Coronary heart disease history (%)					<0.0001
Yes	10.71	6.73	5.88	5.30	
No	88.72	93.06	93.67	93.91	

Mean + SD for continuous variables: P-value was calculated by weighted linear regression model.

% for Categorical variables: P-value was calculated by weighted chi-square test.

## Results

### Baseline characteristics of participants

Table 1 shows the baseline characteristics of participants chosen from NHANES 2003 to 2010 by quartile of serum albumin. Variates including aspartate aminotransferase, uric acid, and triglycerides had no statistically significant differences between different groups. Participants with lower serum albumin were older and had lower cholesterol, poverty-to-income ratio, alanine aminotransferase, higher serum glucose, body mass index, and creatinine compared with the Q4 group. Moreover, participants with decreased serum albumin were more likely to have hypertension, failing kidneys, diabetes, stroke, asthma, emphysema, and coronary heart diseases when compared to the Q4 group. Most of the participants were Non-Hispanic White with a degree above that of high education.

### Univariate and multivariate analysis

Table 2 shows the univariate and multivariate linear regression results. According to the non-adjusted model, the PSA levels decreased with serum albumin levels, decreasing −0.023 (−0.034, −0.013). The relationship between serum albumin and PSA levels was not significant after adjusting for demographic variables (minimally-adjusted) (*p* = 0.21719) and adjusting for all covariates in Table 2 (*p* = 0.09643).

**Table 2:** Univariate and multivariate analysis by weighted linear regression model.

**Exposure**	**Non-adjusted**	**Adjust I**	**Adjust II**
Serum albumin (g/L)	−0.023 (−0.034, −0.013) 0.00002	0.006 (−0.004, 0.017) 0.21719	−0.009 (−0.020, 0.002) 0.09643
**Serum albumin (g/L)**			
Q1	0	0	0
Q2	−0.05 (−0.15, 0.05) 0.3587	0.07 (−0.02, 0.17) 0.1472	0.01 (−0.08, 0.11) 0.7590
Q3	−0.08 (−0.17, 0.02) 0.1130	0.10 (0.01, 0.20) 0.0279	0.01 (−0.09, 0.10) 0.8946
Q4	−0.21 (−0.30, −0.11) < 0.0001	0.04 (−0.05, 0.13) 0.4004	−0.08 (−0.18, 0.01) 0.0780
*P* for trend	<0.001	0.489	0.226

Non-adjusted model adjust for: None.

Adjust I model adjust for: Age; Race/Hispanic; Education Level; Marital status; Poverty income ratio.

Adjust II model adjust for: Race, Education level, Diabetes history, Failing kidney history, Emphysema history, Stroke history, Marital status history, Hypertension history, Asthma history, Coronary heart disease history; Age (year), Poverty income ratio (PIR), Serum glucose (mmol/L), Body mass index (kg/m^2^), Creatinine (umol/L), Aspartate aminotransferase (U/L), Alanine aminotransferase (U/L), Cholesterol (mmol/L), Blood urea nitrogen (mmol/L), Lactic dehydrogenase (U/L), Serum uric acid (umol/L), Triglycerides (mmol/L).

To investigate whether there is a non-linear relationship between serum albumin and PSA, we translated serum albumin into categorical variables by quartile, and trend estimation for serum albumin was performed in a sensitivity analysis (Table 2). Consequently, we found that the trend of effect values was non-isometric among the serum albumin groups. These findings indicate the possibility of non-linearity in serum albumin and PSA levels.

### Identification of non-linear relationship

**Figure 2:**
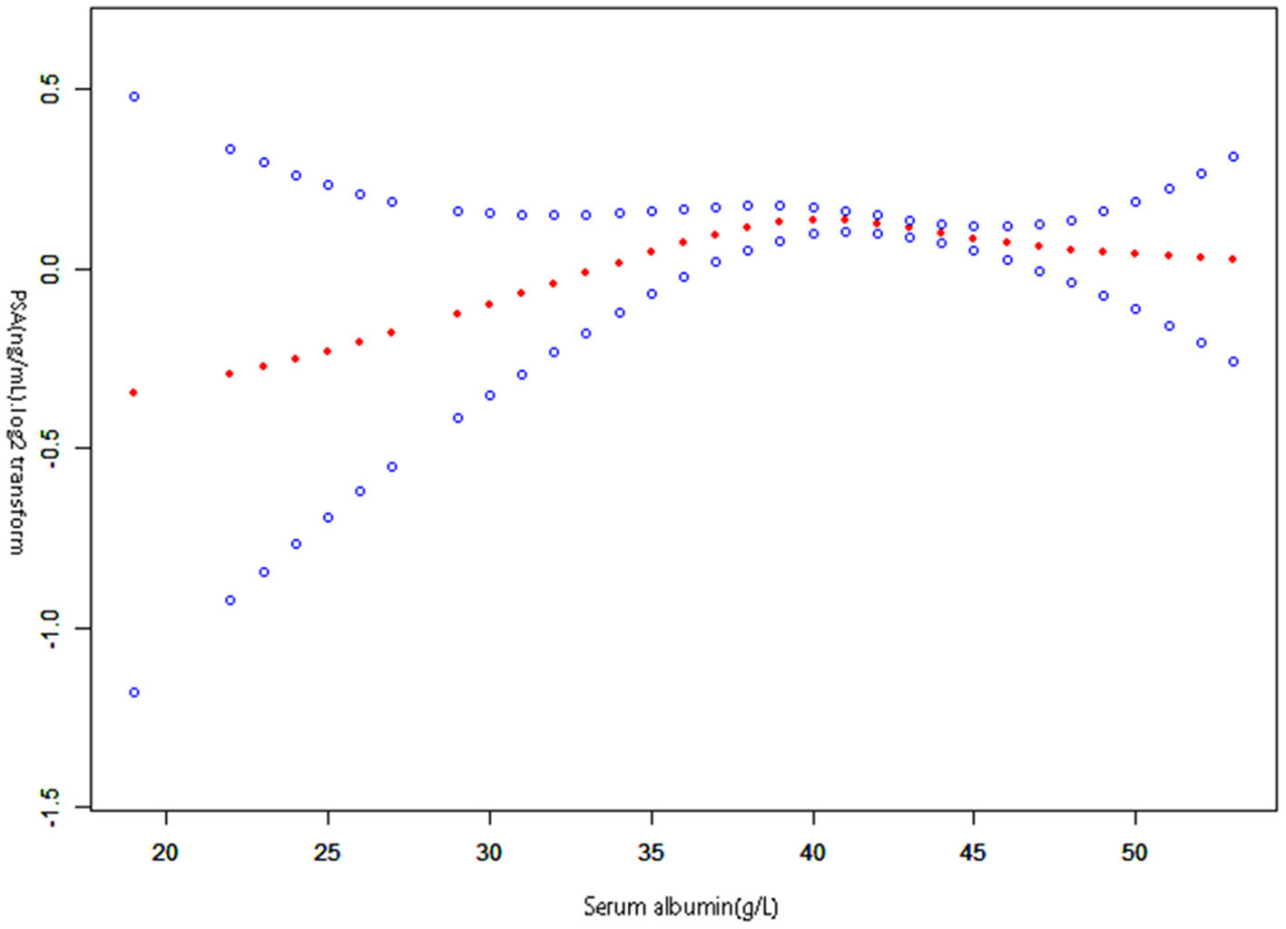
The relationship between serum albumin and prostate-specific antigen. A non-linear relationship between them was detected after adjusting for Race, Education level, Diabetes history, Failing kidney history, Emphysema history, Stroke history, Marital status history, Hypertension history, Asthma history, Coronary heart disease history; Age (year), Poverty income ratio (PIR), Serum glucose (mmol/L), Body mass index (kg/m^2^), Creatinine (umol/L), Aspartate aminotransferase (U/L), Alanine aminotransferase (U/L), Cholesterol (mmol/L), Blood urea nitrogen (mmol/L), Lactic dehydrogenase (U/L), Serum uric acid (umol/L), Triglycerides (mmol/L).

This study examined the non-linear relationship between serum albumin and PSA (Figure 2). Using the smooth curve fitting, we found a non-linear relationship between serum albumin and PSA. We compared a linear regression model with a two-piecewise linear regression model and found *P* equal to 0.013 for the log-likelihood ratio test. This implies that the two-piecewise linear regression model should be applied to the model. By a two-piecewise linear regression model and recursive algorithm, the inflection point was calculated as 41 g /L (Table 3). Left of the inflection point, the effect size, 95%CI, and *P*-value were 0.019 (log2 transformation) (−0.006, 0.043) and 0.1335, respectively. A negative association was noted between serum albumin and PSA on the right side of the inflection point, with effect size, 95%CI, and a *P*-value of −0.022 (log2 transformation) (−0.037, −0.007), 0.0036. Serum albumin and PSA levels demonstrated a somewhat U-shaped relationship with a serum albumin threshold of 41 g/L. These results indicate that serum albumin and PSA levels had a threshold effect.

**Table 3:** Non-linearity addressing by weighted two-piecewise linear model.

**Outcome**	**PSA(ng/ml) log2 transform β (95% CI)**
Fitting by weighted linear regression model	−0.009 (−0.020, 0.002) 0.0964
**Fitting by weighted two-piecewise linear regression model**	
Inflection point	41
<41	0.019 (−0.006, 0.043) 0.1335
>41	−0.022 (−0.037, −0.007) 0.0036
Log likelihood ratio test	0.013

PSA, prostate-specific antigen; Independent variable is serum albumin(g/L) and dependent variable is PSA (ng/mL log2 transform).

Covariates involved in this model was the same as Adjust II model presented in Table 2.

## Discussion

The work investigated the relationship between serum albumin and PSA in a non-PCa population aged over 40 years in the United States. Despite the lack of a linear relationship, our findings revealed that serum albumin negatively correlated with PSA levels when it exceeded 41 g/L.

Several studies have reported relationships between serum albumin and PCa. For instance, Takehiro Sejima et al. discovered that low levels of serum albumin preoperatively may indicate extensive disease in clinically localized prostate cancer and may be linked to biochemical disease recurrence. A low serum albumin level was thought to induce a decrease in albumin-binding testosterone, increasing free testosterone, which was thought to be important for hormone-sensitive PCa (16). Elsewhere, Wang et al. discovered that an increased fibrinogen level and decreased albumin level might contribute to cancer progression and poor outcomes (17). Low albumin levels were indicative of malnutrition and inflammation within the body. There was a high prevalence of malnutrition among cancer patients, with many negative effects including impaired immune function, lessened response to the treatment of cancer, and shorter survival time (18, 19). In inflammatory responses to the tumor or from the tumor itself, several inflammatory mediators were discharged, containing interleukin-1(IL-1), IL-6, necrosis factor, and acute phase reactants, which might promote albumin escape from capillaries and modulate the production of albumin in the liver (20, 21). Consequently, the albumin level may be an indicator of cancer prognosis.

In the US, PSA screening for early prostate cancer detection has become popular over the past 25 years (22). Early detection by PSA testing and appropriate treatment reduces the risk of death for men with aggressive prostate cancer (23). Nonetheless, PSA levels are affected by numerous factors, which have received significant research attention. Wang et al. noted that men with higher liver fibrosis scores had lower serum PSA. Liver dysfunction on a chronic basis can reduce testosterone levels, causing reduced PSA production (24). Song et al. investigated the correlation between PSA and dietary protein intake and found a non-linear correlation. A positive association was observed between dietary protein intake and elevated PSA levels as dietary protein intake exceeded 181.8 g (14). IGF-1 might be the important factor in the relationship between dietary protein intake and PSA concentrations by modifying IGF-1 levels and inhibiting PI3K/AKT/mTOR pathways. Additionally, dietary protein might decrease the sensitivity of insulin and boost the growth of prostate cancer cells in animal models, which in turn affects PSA levels (6, 25, 26). However, the relationship between serum albumin and PSA remains unknown; thus this secondary analysis based on the NHANES data aims to explore it. In our research, we found a non-linear relationship between serum albumin and PSA levels. Moreover, a negative correlation was present between serum albumin and PSA when serum albumin exceeded 41 g/L. The possible mechanism is that increased serum albumin level increased albumin-binding testosterone, resulting in a decrease in free testosterone, which was converted to dihydrotestosterone and regulated prostate development (16). Further studies are necessary to investigate the possible relevant mechanisms.

This study has a number of strengths. First, it has a large sample size, i.e., 5,469 participants, which provides a high level of statistical power to quantify the relationship between PSA and serum albumin. Secondly, we performed both linear and non-linear regression models to increase comparability, and the results indicated a possible non-linear relationship. Finally, a recursive algorithm was used to determine the inflection point, and a two-piecewise linear regression was used to determine the saturation effect of serum albumin and PSA.

On the other hand, this work also has limitations. First, we could not obtain a causal link between PSA and serum albumin due to its cross-sectional design. Secondly, the findings of this work are generally limited since it only focused on the American population. Thirdly, being a secondary analysis of published data, variables not contained in the dataset cannot be regulated.

## Conclusion

In conclusion, serum albumin and PSA have a non-linear relationship. Serum albumin negatively correlates with PSA levels when serum albumin exceeds 41 g/L. However, our findings should be validated through a methodologically robust and large prospective clinical trial.

## Data availability statement

The original contributions presented in the study are included in the article/supplementary material, further inquiries can be directed to the corresponding authors.

## Ethics statement

NHANES protocol was approved by the NCHS Research Ethics Review Board. The patients/participants provided their written informed consent to participate in this study. Written informed consent was obtained from the individual(s) for the publication of any potentially identifiable images or data included in this article.

## Author contributions

KX, CC, and JZh conceived and designed the manuscript, performed statistical analysis, and had primary responsibility for final content. JZe and YL wrote and revised the paper and re-analysis the data for the revised version. ZC drafted the manuscript. KX and SL constructed and cleared data. All authors read and approved the final manuscript.
